# TBC1D14 inhibits autophagy to suppress lymph node metastasis in head and neck squamous cell carcinoma by downregulating macrophage erythroblast attacher

**DOI:** 10.7150/ijbs.68992

**Published:** 2022-02-07

**Authors:** Tao Lu, Yanshi Li, Min Pan, Dan Yu, Zhihai Wang, Chuan Liu, Guohua Hu

**Affiliations:** Department of Otorhinolaryngology, the First Affiliated Hospital of Chongqing Medical University, 1#Youyi Road, Yuzhong District, Chongqing, 400016 China

**Keywords:** head and neck squamous cell carcinoma, lymph node metastasis, TBC1D14, macrophage erythroblast attacher, autophagy

## Abstract

**Aims:** This study aimed to identify the correlation and molecular mechanism between TBC1 domain family member 14 (TBC1D14) and lymph node metastasis (LNM) in head and neck squamous cell carcinoma (HNSCC).

**Methods:** Whole transcriptome sequencing of HNSCC tissues with or without LNM was performed. TBC1D14 expression was quantified in HNSCC tissues. The role of TBC1D14 in HNSCC migration, invasion, autophagy, and LNM was investigated by wound healing, Transwell, western blotting, immunofluorescence, and transmission electron microscopy assays in vitro and in a mouse model in vivo. The correlation between autophagy and LNM was detected by wound healing and Transwell assays in vitro and western blotting in vivo. Mass spectrometry was used to identify the downstream target proteins. The correlation between TBC1D14 expression and macrophage erythroblast attacher (MAEA) expression was identified by qRT-PCR and western blotting assays in vitro and immunohistochemistry in vivo. The gain-of-function strategy was applied to further reveal the role of MAEA in the TBC1D14-induced autophagy of HNSCC cells.

**Results:** TBC1D14 was a co-differentially expressed gene in the sequencing results, The Cancer Genome Atlas Data Portal, and Gene Expression Omnibus databases. TBC1D14 had a lower RNA and protein expression in HNSCC with LNM samples and was a favorable prognostic indicator. TBC1D14 inhibited the migration and invasion of HNSCC in vivo. Mechanistically, TBC1D14-induced autophagy suppression inhibited the migration and invasion of HNSCC. TBC1D14 expression negatively correlated with MAEA expression both in vitro and in vivo. Furthermore, MAEA overexpression could reverse TBC1D14-induced autophagy suppression.

**Conclusion:** TBC1D14 is a novel LNM inhibitor in HNSCC and a favorable prognostic marker. TBC1D14 suppresses autophagy to inhibit LNM in HNSCC by downregulating MAEA expression. The results clarify the molecular mechanism of TBC1D14 in HNSCC.

## Introduction

Head and neck squamous cell carcinoma (HNSCC), which ranks sixth among the most common cancer worldwide, leads to more than 300,000 deaths annually, and the five-year overall survival (OS) is only around 30% 1, 2. Lymph node metastasis (LNM), one of the leading causes of death in HNSCC, could induce the relapse of tumors and reduce the response rate to therapy 3-5. Recently, a multicenter epidemiology study in the USA showed that patients with LNM had an approximate 50% lower five-year OS than those without 1. Despite several attempts to identify biomarkers related to LNM in HNSCC 6-10, not much is known about the molecular mechanism of LNM 11, 12. Moreover, patients with LNM have not benefited much from advances in therapy 2, 13. Thus, understanding the mechanism of LNM is extremely important to improve the outcomes of HNSCC.

Whole-transcriptome sequencing is a new tech that allows for a better understanding of the underlying mechanisms in malignant behaviors of cancer 14, 15. Thus, we performed this sequencing in HNSCC tissues with or without metastasis, then compared the results with other sequencing data from The Cancer Genome Atlas (TCGA) Data Portal and Gene Expression Omnibus (GEO) databases. A novel metastasis suppressor, TBC1 domain family member 14 (TBC1D14), was identified as a co-differentially expressed gene (co-DEG) in the above three cohorts. TBC1D14 belongs to the TBC1 domain-containing protein family, the members of which are reported to function mainly in regulating intracellular trafficking 16, 17. Despite several recent studies reporting members of the TBC1 domain family as tumor suppressors in breast cancer and epithelial ovarian cancer 18-21, the exact role of TBC1D14 in HNSCC remains poorly understood. Therefore, it is necessary to reveal the key role and detailed mechanism of TBC1D14 in LNM occurrence in HNSCC.

Autophagy (here referred to as macroautophagy), characterized by the formation of double-membrane vesicles, is an evolutionarily intracellular conserved process by which cells can degrade cytoplasmic components to generate nutrients in response to stress conditions 22, 23. The pro-metastasis role of autophagy in colorectal cancer 24, gastric cancer 25, breast cancer 26, 27, and prostate cancer 28 has been recognized. Autophagy helps tumor cells escape from anoikis (a special type of apoptosis induced by the loss of attachment to the extracellular matrix) and survive at the sites of metastasis in response to hypoxia, nutrient deprivation, and reactive oxygen species 29. Autophagy was also shown to be closely related to epithelial-mesenchymal transition (EMT) 30, 31, which is considered a key process to promote cancer metastasis. These studies together elucidated that autophagy could be partially attributed to the metastasis of tumor cells. Two studies have revealed that TBC1D14 suppresses autophagy in 293T cells 32, 33. However, the role of TBC1D14 in tumor cells has not yet been elucidated. Considering the pro-metastatic role of autophagy in tumors, we hypothesized that TBC1D14 inhibits the metastasis of HNSCC through the suppression of autophagy.

This study was conducted to provide insights into exploring the biological markers of LNM in HNSCC and elucidating the underlying mechanism to better inform treatment decisions. With the help of whole-transcriptome sequencing, the novel anti-metastatic role of TBC1D14 in HNSCC was identified. Moreover, TBC1D14 was found to suppress autophagy by downregulating macrophage-erythroblast attacher (MAEA). TBC1D14 is a potential favorable biomarker and therapeutic target of LNM in HNSCC.

## Materials and methods

### Tissue collection

A total of 74 patients with HNSCC who underwent surgeries at the First Affiliated Hospital of Chongqing Medical University (Chongqing China) between 2014 and 2019 were enrolled. Patients who received radiotherapy and/or chemotherapy before surgeries were excluded. The protocol was approved by the institutional ethics committee of the First Hospital of Chongqing Medical University, and all the patients provided written informed consent.

### Microarray analyses

The library construction and sequencing were performed by Sinotech Genomics Co., Ltd. (Shanghai, China). Total RNA of six tumor tissues (three cases with and three cases without LNM) was extracted from fresh frozen samples using the RNeasy Mini kit (Cat. No. 74106, Qiagen, Hilden, Germany), after which the integrity and the concentration were tested with the help of Agilent BioAnalyzer 2100 (Agilent Technologies, Santa Clara, USA) and Qubit^®^ 3.0 Fluorometer (Thermo Fisher, Waltham, USA). The whole transcriptome of the above RNA was then sequenced using the Illumina NovaSeq 6000 platform in the paired-end mode. Genes with a fold change ≥ 1.5 and a false discovery rate (FDR) < 0.05 were defined as differentially expressed genes (DEGs).

### Quantitative real-time PCR (qRT-PCR)

Total RNA of fresh frozen HNSCC tissues (10 cases with LNM and 10 cases without LNM) or cell lines were obtained using the E.Z.N.A.^®^ Total RNA kit I (Omega Bio-Tek, Norcross, USA), and the cDNA was then reverse transcribed using PrimeScript RT reagent kit (Takara, Dalian, China). qRT-PCR was performed using SYBR® Green (Takara, Dalian, China) according to the manufacture's protocol. The relative mRNA expression was calculated using the 2-ΔΔCt method, while GAPDH was set as an internal control. Sequences of the primers are listed in Supplementary Table S1.

### Protein extraction and western blotting

Tumor tissues or cell lines were lysed using the whole-cell lysis assay kit (KeyGEN BioTECH, Nanjing, China) to obtain total protein according to the standard protocol. Western blotting analysis was performed according to the standard protocol. The details of antibodies are described in Supplementary Table. S2.

### Immunohistochemistry (IHC)

Continuous sectioned slides (5 μm) obtained from specimens that were previously fixed with 10% formalin solution and embedded with paraffin were stained using an immunohistochemistry kit (ZSGB-BIO, Beijing, China). In brief, xylene and graded ethanol were applied to de-paraffinize the slides, while sodium citrate buffer (0.01 M, pH 6.0) was used to perform antigen retrieval for 20 min at 100℃. All sections were blocked with endogenous peroxidase blockers at 37℃ for 15 min before incubation with primary antibodies at 4℃ overnight. After the reaction with biotin-labeled goat anti-rabbit secondary antibodies for 20 min at 37℃, all sections were washed with phosphate buffer saline (PBS) three times and visualized after staining with diaminobenzidine and hematoxylin. Image-Pro Plus software (version 6.0; Media Cybernetics, Rockville, USA) was applied to measure the mean integrated optical density of each section. Based on the staining intensity and range, the sections were dichotomized into two groups: positive expression (stained brown, and stained tumor cells were no less than 50%) and negative expression (stained yellow, and stained tumor cells were less than 50%).

### Cell lines and transfection

HNSCC cell lines (Fadu and SCC15) were purchased from the cell bank of the Chinese Academy of Sciences (Shanghai, China). Cells were cultured in Dulbecco's modified Eagle's medium (DMEM; Gibco, Middletown, USA) containing 10% fetal bovine serum (Gibco, Australia origin) and 1% penicillin/streptomycin (Beyotime, Shanghai, China) in a humidified atmosphere at 37°C with 5% CO_2_.

GV344 (hU6-MCS-Ubiquitin-firefly_Luciferase-IRES-puromycin) vector (Genechem, Shanghai, China) containing short-hairpin RNA (shRNA), which targeted human TBC1D14 (sequence listed in Supplementary Table. S1), was applied to construct the shTBC1D14 plasmid. GV260 (Ubi-firefly_Luciferase-IRES-Puromycin) vector (Genechem, Shanghai, China) containing human TBC1D14 coding gene was used to establish the TBC1D14 overexpression plasmid. GV344 and GV260 vectors without other sequences were set as negative controls. All the plasmids were then coated with lentivirus (LV). HNSCC cells were first plated in six-well plates at a density of 1×10^6^ cells/well. When the cell density of the culture reached about 50%, 1 ml medium with the corresponding LV and 40 µl Transfection reagent A (Genechem, Shanghai, China) were added to each well. After 24 h, the transfection medium was replaced by 2 ml DMEM with 10% fetal bovine serum. When the cells reached about 80% density, 2 ml medium with puromycin (2 µg/ml) was added; wild-type cells were set as control. After 48 h, the concentration of puromycin was reduced to 0.5 µg/ml when wild-type cells were almost dead. After a one-month screening period, the cell line with stable TBC1D14 overexpression or knockdown (KD) was established, and puromycin was abandoned.

Human MAEA-encoding gene was cloned into the pcDNA3.1 plasmid (TSINGKE Biological Technology, Beijing, China). Cells were first plated in six-well plates at a density of 1×10^6^ cells/well. Then, 2000 ng plasmids and 4 µl Lipofectamine 2000 (Invitrogen, Carlsbad, USA) were mixed in 1.5 ml DMEM and then added into each well when the density of the cells reached about 60%. After 6 h, the transfection medium was replaced by 2 ml DMEM containing 10% fetal bovine serum.

### Wound healing assay

Cells were first seeded into 6-well plates at a density of 1×10^6^ cells/well. When a confluent monolayer of cells formed, an artificial scratch was made using a 200-μl pipette tip. Cells were then cultured in a serum-free medium and maintained at 37℃ in 5% CO_2_. The migration of cells into the wound was observed at 0 and 24 h after the scratch was made via microscopy.

### Transwell migration and invasion assay

Cells were seeded into a 24-well Transwell chamber (Corning, New York, USA). In brief, cells (migration assay: 2×10^4^; invasion assay: 5×10^4^) in 200 µl serum-free medium were added into the upper chamber, and 600 µl DMEM containing 20% FBS was added into the lower chamber. After 48 h of incubation at 37 ℃, cells in the upper chamber were removed and the rest of the cells were first fixed with 100% methanol and then stained with 0.5% crystal violet. Representative pictures were taken through a microscope, and the number of cells was counted in five random fields. For the invasion assay, the chamber was precoated with Matrigel (BD Biosciences, San Jose, USA).

### Immunofluorescence (IF) assay

Cells were seeded in a 24-well plate already containing coverslips. After being treated with DMSO or chloroquine (10 µg/ml), the cells were first fixed with 4% paraformaldehyde for 30 min, incubated with 0.1% TritonX-100 for 15 min, blocked with goat serum (Beyotime, Shanghai, China) for 1 h at room temperature, then incubated with primary antibodies at 4℃ overnight. After washing with PBS thrice, the cells were incubated with the corresponding secondary antibodies for 1 h and DAPI (Beyotime, Shanghai, China) for 10 min. Imagines were obtained using a confocal laser scanning microscope (Carl Zeiss AG, Oberkochen, Germany). The details of antibodies are described in Supplementary Table. S2.

### Transmission electron microscopy (TME)

Cells previously treated with DMSO or chloroquine (10 µg/ml) were collected, fixed in 2.5% glutaraldehyde at 4℃ overnight, treated with 1% osmium tetroxide, then dehydrated in graded ethanol, series before being embedded in Epon. Finally, the slides were stained with uranyl acetate/lead citrate. Representative images were obtained using a transmission electron microscope (Hitachi, Ltd, Tokyo, Japan).

### Sample preparation and mass spectrometric analysis

Cells were collected and lysed using moderate lysis buffer (2% SDS, 7M Urea). After centrifugation at 13000 x rpm for 20 min at 4 °C, six times volume of 100% acetone was added and precipitated overnight at -20 ℃. Peptides were reconstituted according to the manufacture's protocol of filter-aided sample preparation (FASP) and then were desalinated with a Monospin column. Finally, samples were subjected to mass spectrometry with Orbitrap Fusion Lumos (Thermo Scientific, Waltham, USA) setting at Data Dependent Acquisition (DDA) mode.

### Hematoxylin and eosin (H&E) staining

Fixed and embedded tissues from mice were sequentially sectioned into 5 µm slides and were de-paraffinized with xylene and graded ethanol. After washing with PBS three times, the slides were first stained with hematoxylin for 1 min, incubated with differentiation fluid (1% hydrochloric acid alcohol) for 1 min, then stained with eosin for 2 min, and finally dehydrated with graded ethanol and xylene. Images were obtained through a microscope at 100X and 400X magnifications.

### Popliteal lymph node metastasis (LNM) model

Approximately 5×10^6^ LV-infected Fadu cells (Ubi-MCS-firefly_Luciferase-IRES-Puromycin) in 50 µl PBS were inoculated subcutaneously into the left footpads of nude mice (5 mice/group). Tumor growth was monitored every 5 days, and tumor volume was calculated as previously described 34. Forty days after inoculation, the mice were intraperitoneally injected with D-Luciferin potassium salt (Beyotime, Shanghai, China) at a dose of 150 µg/g and then imaged using the In Vivo Imaging System (IVIS; Berthold Technologies, Baden Wurttemberg, Germany). The mice were then sacrificed, and the tumors and lymph nodes (LNs) were excised, weighed, and embedded in paraffin for HE and IHC detection. All study protocols were approved by the Animal Care and Treatment Committee of Chongqing Medical University.

### Bioinformatics analysis

The transcriptome and the corresponding clinical data of HNSCC datasets were downloaded from TCGA Data Portal and GSE117973 datasets in GEO databases. By comparing patients with or without LNM, we identified DEGs using the edgeR package. DEGs were genes with a fold change ≥ 1.5 and FDR < 0.05. A heatmap of co-DEGs was generated using the pheatmap package, and the corresponding Venn diagram was plotted using the Draw Venn Diagram website (http://bioinformatics.psb.ugent.be/webtools/Venn/).

### Data analysis

All analyses for statistical differences were performed using SPSS software (Version 22.0; SPSS Inc, Richmond, USA), and a difference with a P-value < 0.05 was defined as significant. The significance in data from qRT-PCR, western blotting, wound healing, Transwell, immunofluorescence, and transmission electron microscopy was analyzed using the Student's t-test. The correlation between the expression of TBC1D14 and clinicopathological characteristics was determined using the Pearson χ2 test. The survival curve was plotted using the Kaplan-Meier method with a log-rank test for p-value. The workflow graphs were generated using BioRender (https://biorender.com/).

## Results

### Transcriptomic profiles revealed the association between TBC1D14 and clinicopathological characteristics of HNSCC

**Fig 1:**
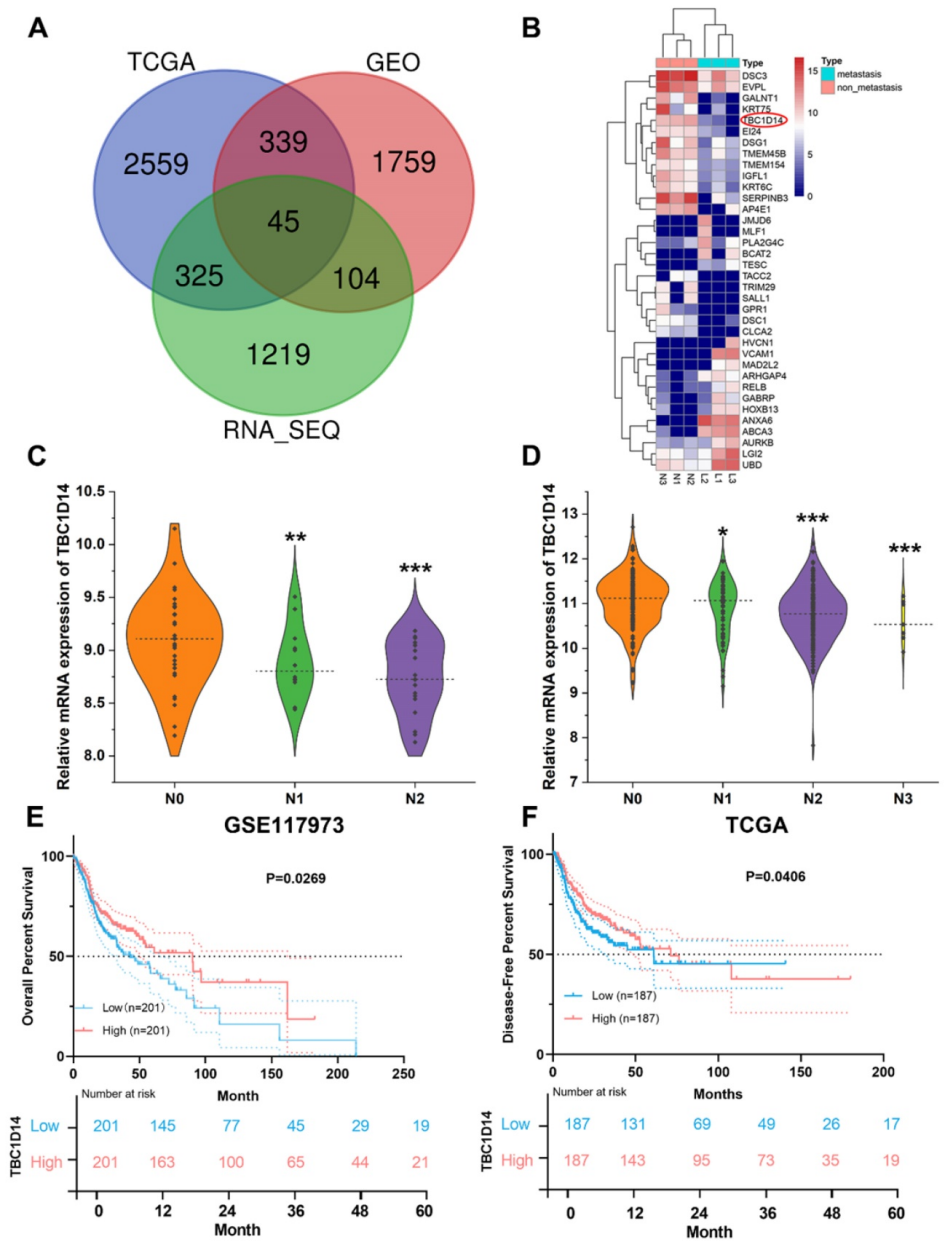
** Negative correlation between TBC1D14 expression and lymph node metastasis (LNM) was identified by RNA-sequencing in head and neck squamous cancer (HNSCC) patients.** (A) A total of 45 co-differentially expressed genes (co-DEGs) were identified between HNSCC patients with and without LNM. (B)Total co-DEGs with the same tendency in our whole transcriptome sequencing were displayed by heatmap. (C-D) The expression of TBC1D14 was negatively correlated with the N stage in patients from both GEO datasets (C) and TCGA datasets (D). (E-F) TBC1D14 was a favorable prognostic marker both in overall survival (E) and disease-free survival (F) in data from TCGA datasets. * *P*-value <0.05, **0.01; ***0.001. L: primary-site tumors with lymph node metastasis. N: primary-site tumors without lymph node metastasis. ML: metastatic lymph node.

Surgically resected primary tumor tissues from six cases of HNSCC patients (three cases with LNM and three cases without LNM) were selected for whole-transcriptome sequencing analysis and identified 1693 DEGs (upregulated: 914; downregulated: 779). For further validation, the transcriptome and corresponding clinical data of HNSCC datasets were downloaded from TCGA Data Portal, and GSE117973 datasets were obtained from GEO databases. Following the same methods, 3268 DEGs (upregulated: 1907; downregulated: 1361) were generated from TCGA Data Portal, and 2247 DEGs (upregulated: 1146; downregulated: 1101) were identified from GSE117973 datasets. An integrated analysis of these three lists of DEGs revealed 45 co-DEGs (Fig. 1A and Supplementary Table S3), and 36 genes showed the same trend in all the lists. All co-DEGs are shown in Fig.1B. TBC1D14 was one of the co-DEGs, and low expression of TBC1D14 was closely related with more advanced N stage in both GEO (Fig. 1C) and TCGA cohorts (Fig. 1D). Furthermore, stratification of patients from TCGA HNSCC cohort, using median TBC1D14 expression as the cutoff threshold, OS and disease-free survival (DFS) were analyzed in TBC1D14^high^ and TBC1D14^low^ subgroups of patients. TBC1D14^high^ patients displayed significantly improved OS and DFS, as compared to TBC1D14^low^ group (Fig. 1E-1F).

### Low expression of TBC1D14 contributed to LNM in patients with HNSCC

**Fig 2:**
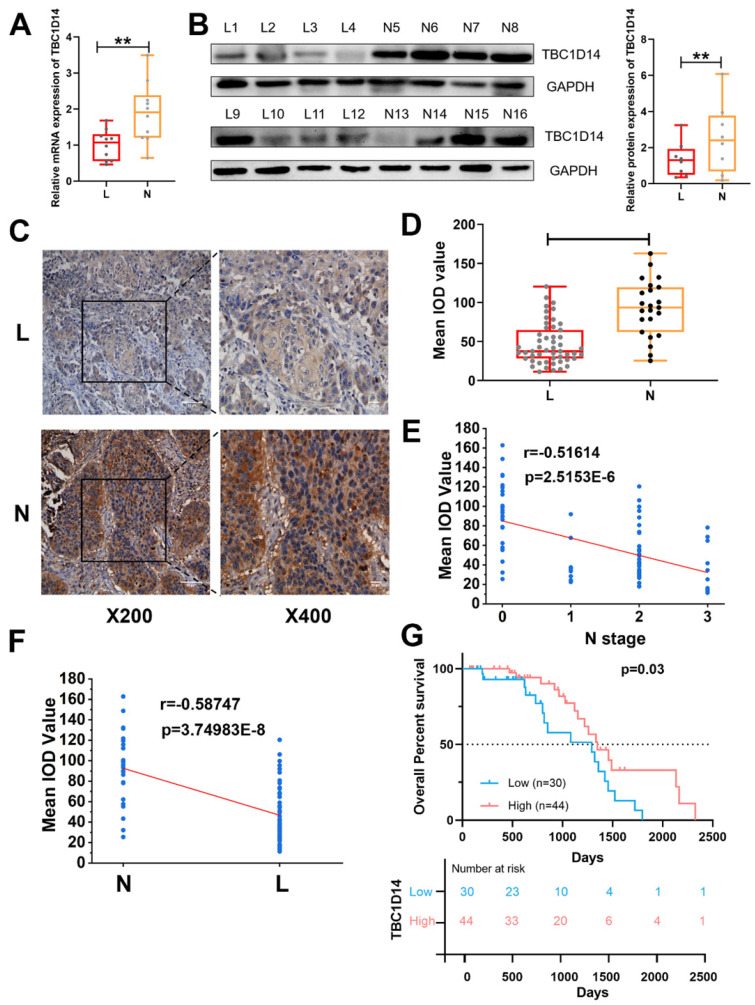
** The expression of TBC1D14 was lower in HNSCC patients with lymph node metastasis.** (A) qRT-PCR analysis of 20 cases of HNSCC primary sites (10 cases with LNM, 10 cases without LNM). (B) The protein level of TBC1D14 was detected in 16 cases of HNSCC primary sites (8 cases with LNM, 8 cases without LNM) by Western blotting analysis. (C) Immunohistochemical staining was performed to reveal the correlation between TBC1D14 and clinical characteristics in 74 cases of HNSCC patients. (D) IOD values of 74 cases of HNSCC tissues. (E-F) The correlation between the expression of TBC1D14 and N stage (E) or metastasis status (F). (G) Kaplan-Meier analysis was applied to reveal the correlation between TBC1D14 expression and overall survival. ** P-value <0.01; ****0.0001. ns: not significantly difference. L: primary-site tumor with lymph node metastasis. N: primary-site tumor without lymph node metastasis. ML: metastatic lymph node.

Considering the aforementioned correlation between TBC1D14 and LNM revealed by TCGA and GEO data analysis, to further test effects of TBC1D14 on lymphatic metastasis, qRT-PCR analysis with 20 HNSCC tissue samples (10 cases with LNM and 10 cases without LNM) and western blotting analysis with 16 HNSCC tissue samples (eight cases with LNM and eight cases without LNM) were performed. As shown in Fig. 2A-2B, low expression of TBC1D14 was more frequent in patients with LNM than in those without LNM both at the mRNA (P = 0.0077) and protein (P = 0.0052) levels. Furthermore, qRT-PCR analysis of 20 HNSCC tissue samples and paired adjacent normal tissues also identified the anticancer role of TBC1D14 (Fig. S1A). Next, expression levels of TBC1D14 were assessed with immunohistochemistry (IHC) on 74 HNSCC tissue samples (51 cases with LNM and 23 cases without LNM) and paired adjacent normal tissues to address the correlation between TBC1D14 expression and clinical parameters (Fig. 2C, Fig. S1B-C, and Table 1). Patients with LNM had a higher incidence of weak staining than those without LNM (38/51 vs. 6/23; P < 0.001), whereas normal tissues showed strong staining. The IHC staining were further quantified using the mean integrated optical density value scoring, which indicated a significantly higher expression level of TBC1D14 in the non-metastasis group as compared to the metastasis group (P < 0.0001; Fig. 2D). Additionally, a significantly negative correlation was observed between TBC1D14 expression and N stage or LNM (r = -0.51614 and -0.58747, respectively; Fig. 2E-2F). As for the correlation between TBC1D14 expression and clinical characteristics, a low expression of TBC1D14 contributed to high N stage (P = 0.001) and extranodal extension (ENE; P = 0.042). Stratification of patients from our HNSCC cohort, using TBC1D14 expression, confirmed similar clinical outcome and poor OS of TBC1D14 low expression patients, comparable with the TCGA cohort (Fig. 2G). Hence, low expression of TBC1D14 is a marker of poor clinical outcome and higher possibility of lymphatic metastasis in HNSCC patients. This prompted us to investigate potential molecular rational for the observed impact of TBC1D14.

### TBC1D14 suppressed the migration and invasion of HNSCC cells

**Fig 3:**
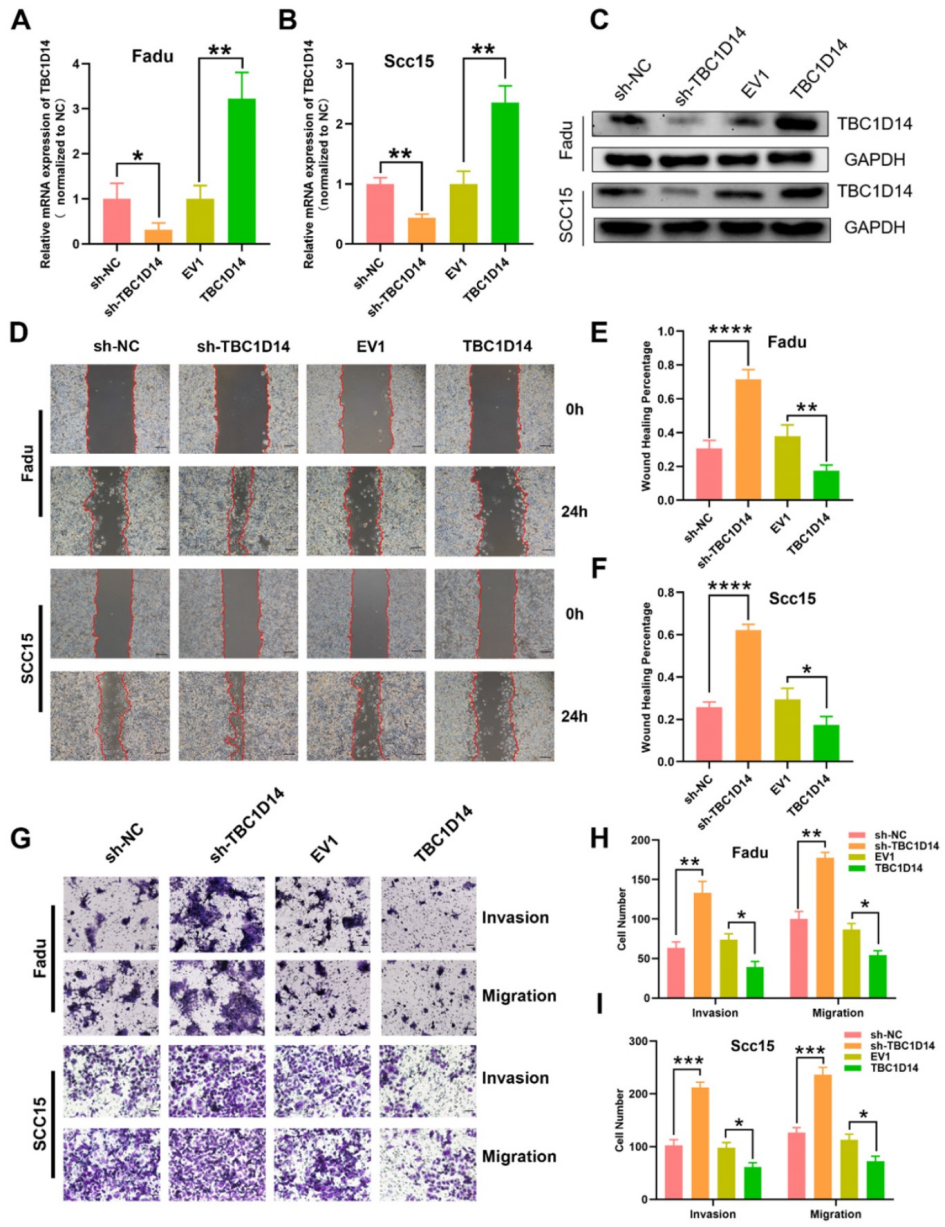
** TBC1D14 inhibited migration and invasion ability of HNSCC cell.** TBC1D14 mRNA level (A) and protein (B) level was measured in Fadu and SCC15 cells with stable expression of empty vector, of TBC1D14 coding vector, of negative control shRNA, and TBC1D14 shRNA. GAPDH was set as a housekeeping gene. (C) Migration assay was conducted to assay the effect of TBC1D14 on the migration ability of HNSCC cells. Pictures were taken at the timeline listed in the diagrams. Scale bar: 100 μm. (D) Wound healing percentage was calculated with ImageJ software from 3 independent experiments. (E) Transwell assay with or without Matrigel was performed to assess the role of TBC1D14 on the invasion and migration ability of HNSCC cells. Scale bar: 100 μm. (F) Graphs showing the numbers of spreading cells from 3 independent experiments. * *P*-value <0.05, **0.01; ***0.001; ****0.0001. NC: negative control, EV1: empty vector of TBC1D14.

In order to address the impact of TBC1D14 expression on tumor cell behavior at the mechanistic level, the stable overexpression or downregulation of LV-mediated TBC1D14 was established with HNSCC cell lines FaDu and SCC15. TBC1D14 expression levels were confirmed by qRT-PCR and western blotting (Fig. 3A-3C). Next, wound healing assay and Transwell assays were conducted to explore the role of TBC1D14 on the migration and invasion abilities of the cells. TBC1D14 overexpression significantly decreased the wound healing rate both in Fadu and SCC15 cells (P = 0.0091 and P = 0.0338, respectively), whereas silencing of TBC1D14 resulted in enhanced wound healing rate (P = 0.0006 and P < 0.0001, respectively; Fig. 3D-3F). In the Transwell assay, the migration and invasion abilities of both HNSCC cell lines were significantly reduced in TBC1D14 overexpression cells, whereas substantially increased migration and invasion were demonstrated following TBC1D14 knockdown (Fig. 3G-3I).

### TBC1D14 inhibited autophagy in HNSCC cells

**Fig 4:**
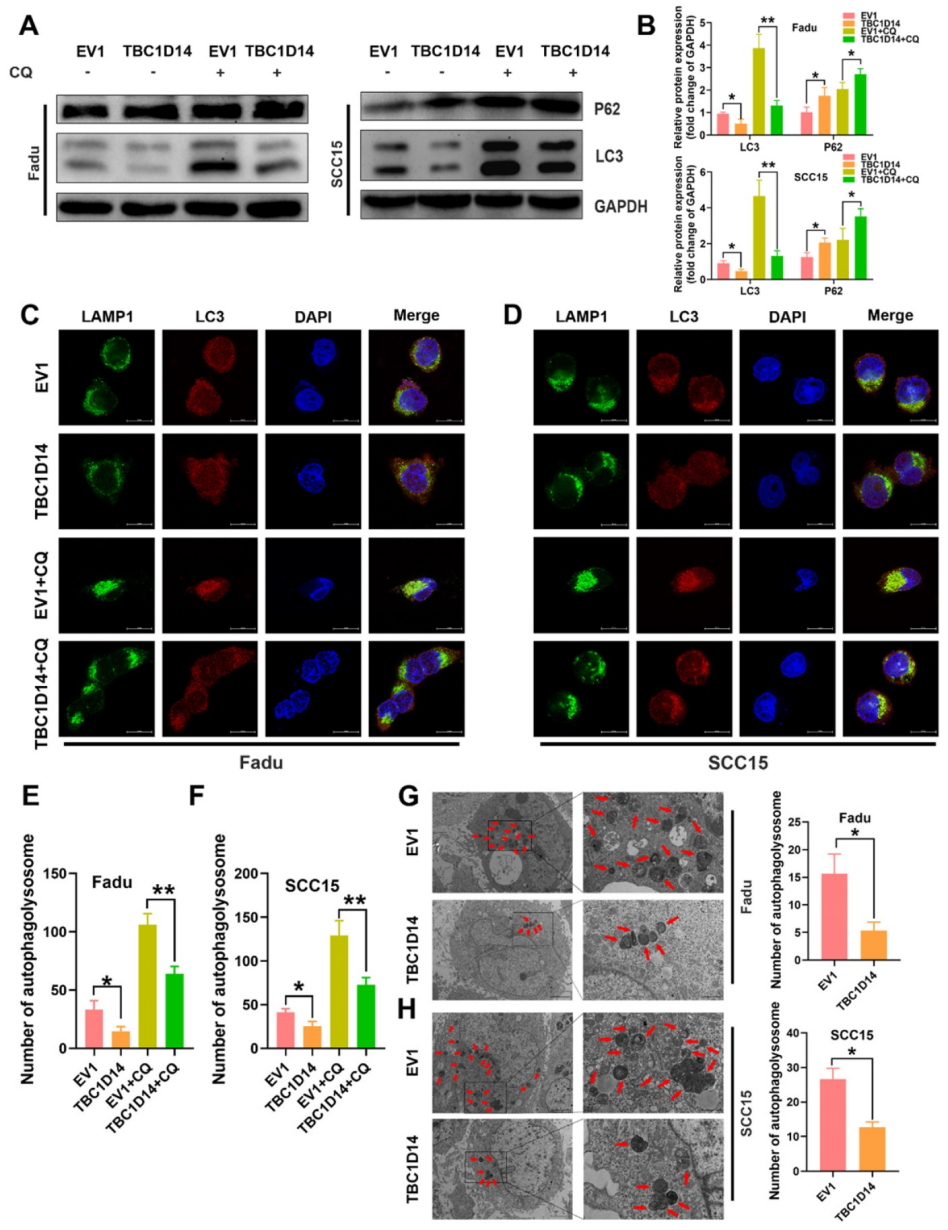
** TBC1D14 exerted inhibited role on HNSCC cell autophagy.** (A-B) The protein level of autophagy markers was detected in TBC1D14-overexpressed cells treated with DMSO or CQ (10μm/ml) for 24h. Strip images were shown in left side and the graphs with calculated ratio were shown in right side. (C-F) Immunofluorescence assay was performed to evaluated the co-localization of LC3 (green) and LAMP1 (red) in TBC1D14-overexpressed fadu (C) and SCC15 (D) cells treated with DMSO or CQ (10μm/ml) for 24h. Co-locablized puncta were counted (E-F) with the help of ImageJ software. scare bar:10μm. (G-H) Autophagosomes were observed with the help of transmission electron microscope in fadu (G) or SCC15(H) cells with empty vector or TBC1D14 coding vector. Red arrows indicated the representative autophagosomes. Counted numbers of autophagosomes were shown in right-side graphs. Scare bar: 5μm (x1500), 2μm (x5000). Each experiment was performed 3 times independently. * *P*-value <0.05, **0.01. CQ: chloroquine. EV1: empty vector of TBC1D14.

Autophagy, induced by nutritional deficiency, is a protective self-degradative process that plays several key roles in promoting the malignant behavior of cancer cells in HNSCC 35-38. Although TBC1D14 was reported to inhibit autophagy in 293T cells 32, 33, 39, 40, whether TBC1D14 could also inhibit autophagy in HNSCC was not revealed. Thus, we firstly investigate the effect of TBC1D14 on autophagy in HNSCC by examining protein markers of autophagy in Fadu and SCC15 cells with stable overexpression of TBC1D14 or empty vector. Western blotting analysis showed that TBC1D14 overexpression could lead to a lower L3C-II/LC3-I ratio and a higher SQSTM1/p62 level (Fig. 4A), which indicated that TBC1D14 affected the autophagy of HNSCC cells. However, it was still unclear whether TBC1D14 could facilitate or inhibit the autophagy flux.

Treatment with chloroquine (CQ), which inhibits the formation and degradation of autolysosome, upregulated the L3C-II/LC3-I ratio to a greater extent in the empty vector group than in the TBC1D14 overexpression group, while SQSTM1/p62 levels were in contrast (Fig. 4A). Taken together, these results revealed the inhibitory effect of TBC1D14 on autophagy in HNSCC. The correlation between TBC1D14 expression and autophagy was further studied via an immunofluorescence assay to detect the colocalization of LC3 (autophagosome) and LAMP1 (lysosome marker). As shown in Fig. 4C-4F, TBC1D14 overexpression significantly decreased the merged yellow puncta with or without CQ treatment, suggesting that TBC1D14 could inhibit the autophagolysosome formation. Transmission electron microscopy 41 revealed that HNSCC cells with TBC1D14 overexpression had fewer numbers of autophagic vacuoles than cells with the empty vector (Fadu: P = 0.0095; SCC15: P = 0.0021; Fig. 4G). These results suggest that TBC1D14 negatively regulates autophagy in HNSCC cells.

### TBC1D14 inhibited the migration and invasion of HNSCC cells via autophagy

**Fig 5:**
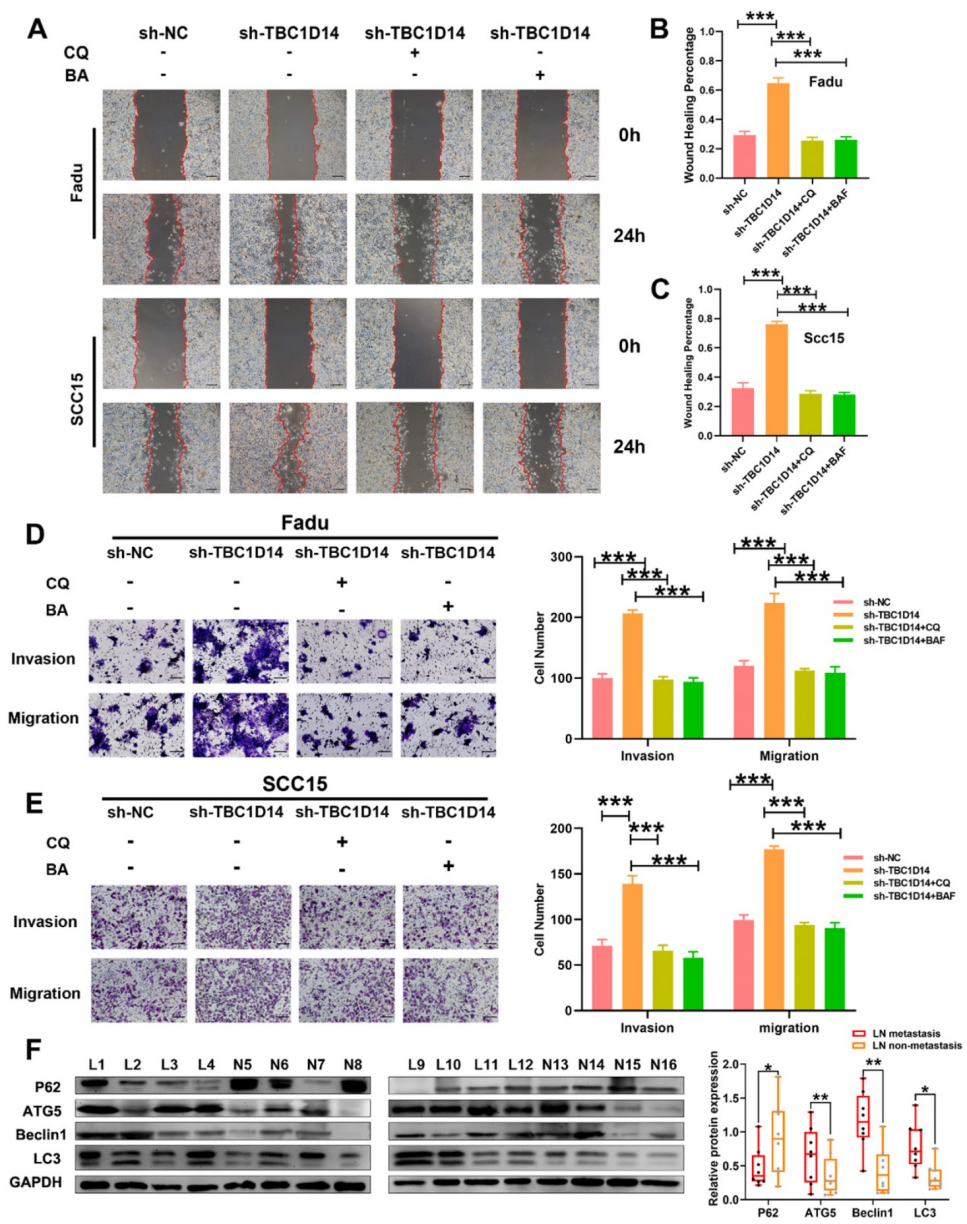
** TBC1D14 suppressed cell migration and invasion through the inhibition of autophagy.** (A-C) Wound healing assay was performed in TBC1D14-downregulated cells treated with DMSO, CQ (10μm/ml), or BA (100nm/ml). Representative images taken at 0h and 24h were shown on the left side. The mean percentage of wound healing area was shown on the right side. Scale bar: 100μm. (D-E) Migration and invasion ability of Fadu (D) and SCC15 (E) cells treated with DMSO, CQ (10μm/ml), or BA (100nm/ml) were analyzed by Transwell assay. Numbers of cells spreading through the transwell were counted by ImageJ software. Scale bar: 100μm. (F) Autophagy markers were detected in 16 cases of HNSCC primary-site tissues (8 cases with and 8 cases without lymph node metastasis) by western blotting analysis. * *P*-value <0.05, **0.01; ***<0.001. CQ: chloroquine. BA: bafilomycin A1. NC: negative control.

Several recent studies have highlighted the role of autophagy in metastasis 26, 30. Given that TBC1D14 could inhibit both autophagy and the migration/invasion ability of HNSCC cells, it was hypothesized that TBC1D14 inhibits the migration/invasion ability of HNSCC cells by suppressing autophagy. To test this hypothesis, TBC1D14-knockdown HNSCC cells were treated with the autophagy inhibitor CQ (10 μm/ml; 24h) or bafilomycin A1 (BA, 100 nm/ml; 24h) to observe the variation in migration and invasion ability. As shown in Fig. 5A-C, CQ and BA treatment significantly reversed the enhanced migration ability caused by the downregulation of TBC1D14 both in Fadu (CQ: P < 0.001; BA: P < 0.001) and SCC15 (CQ: P < 0.001; BA: P < 0.001) cells. Beside on that, the Transwell assay revealed similar results (Fig. 5D-E). Western blotting analysis of primary-site tissues of HNSCC with or without LNM revealed that patients with LNM had a higher protein expression of Beclin1, Autophagy-Related 5 (ATG5), and LC3-II and a lower expression of SQSTM1/p62 (Fig. 5F). Overall, these results confirmed that TBC1D14-mediated autophagy was required for its inhibitory effect on metastasis.

### TBC1D14 targeted MAEA in HNSCC cells

**Fig 6:**
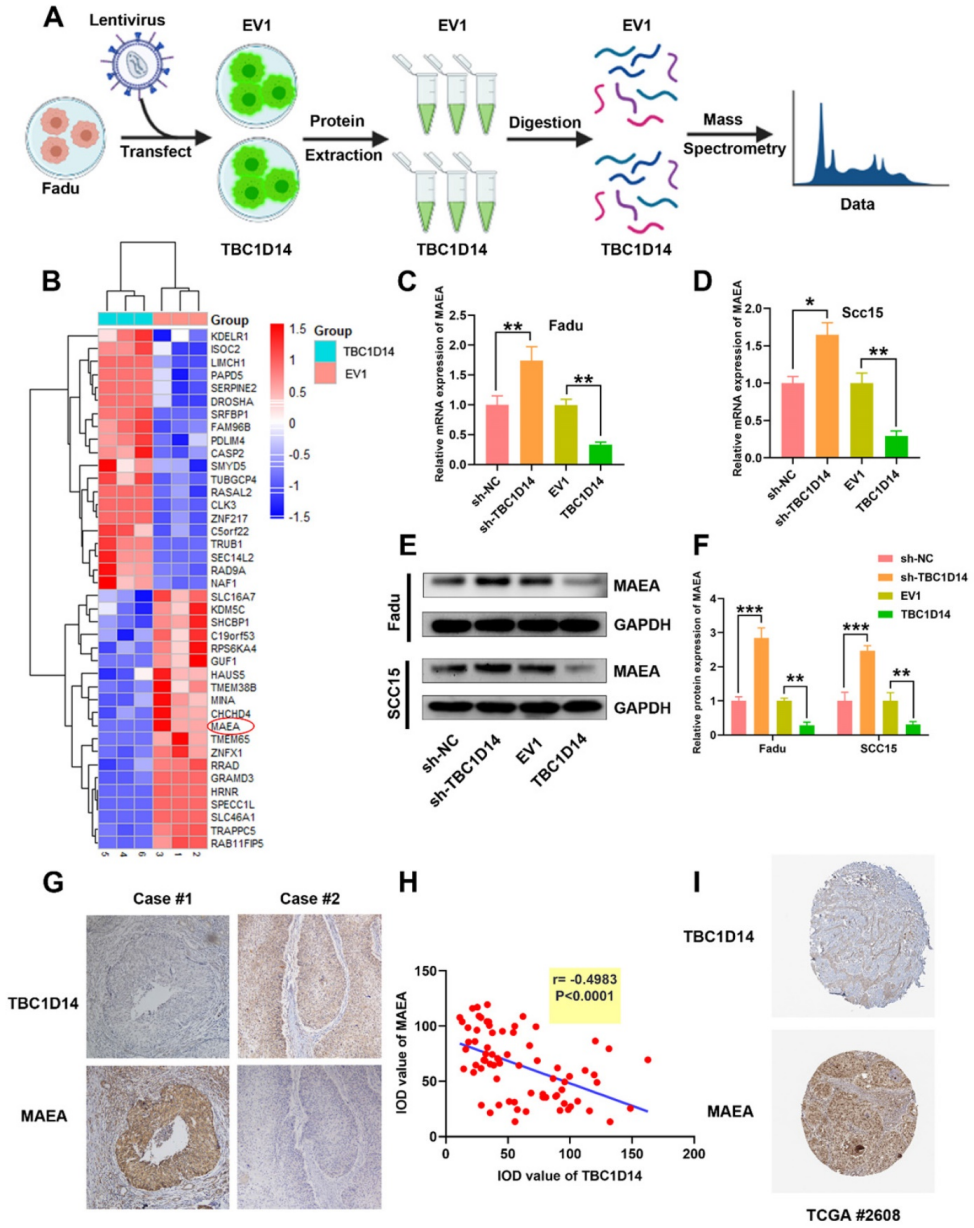
** TBC1D14 downregulated MAEA both in HNSCC Cell line and HNSCC tissues.** (A) The flow diagram of proteomics analysis. (B) TOP 20 down- or up-regulated proteins were shown in the heatmap. (C-F) The correlation between TBC1D14 and MAEA expression in HNSCC cell line was performed with qRT-PCR analysis (C-D) and western blotting analysis (E-F). (G-H) Immunohistochemical staining was performed to detect the correlation between TBC1D14 and MAEA expression in HNSCC tissues. Representative pictures were shown on the left side (G) and results of correlation analysis were placed on the right side (H). (I) Validation of the correlation between TBC1D14 and MAEA was performed in Human Protein Atlas datasets. * *P*-value <0.05, **0.01. EV1: empty vector of TBC1D14. TCGA: The Cancer Genome Atlas.

So far, we have shown that TBC1D14 reduces the migration and invasion ability of HNSCC cells via inhibiting autophagy. In order to shed light on signaling pathways implicated in TBC1D14-mediated inhibition, proteomics analysis was performed on Fadu cells stably overexpressing TBC1D14 and empty vector (Fig. 6A) and 248 differentially expressed proteins (DEPs) were identified, including 153 upregulated and 95 downregulated proteins. The top 20 upregulated or downregulated DEPs are shown in Fig. 6B. Given the significant inhibitory effect of TBC1D14 on autophagy, it was hypothesized that TBC1D14 downregulates the levels of proteins involved in autophagy or upregulates the levels of proteins inhibiting autophagy. To verify the hypothesis, the levels of the top 20 autophagy-related proteins were quantified. MAEA, one of the top 10 down-regulated proteins, has been reported to promote autophagy in brain cells, fibroblasts, hepatocellular carcinoma cells 42-44 and thus was identified as the potential target. As shown in Fig. 6C-6F, the upregulation of TBC1D14 significantly reduced both the mRNA and protein levels of MAEA. In contrast, TBC1D14 knockdown could reverse the downregulation of MAEA caused by TBC1D14 both at the mRNA and protein levels. The correlation of TBC1D14 expression and MAEA in HNSCC tissues was further analyzed in 74 cases of HNSCC tissues using IHC staining. Interestingly, a significantly negative correlation was identified between the expression of TBC1D14 and MAEA (r = -0.4983, P < 0.0001; Fig. 6G). Downloaded data from Human Protein Atlas datasets (HPA) confirmed the negative correlation of TBC1D14 and MAEA in HPA HNSCC cohorts (Fig. 6H). Collectively, these findings suggested that TBC1D14 downregulates the expression of MAEA in HNSCC.

### MAEA overexpression reversed the TBC1D14-medicated inhibition of autophagy in HNSCC cells

**Fig 7:**
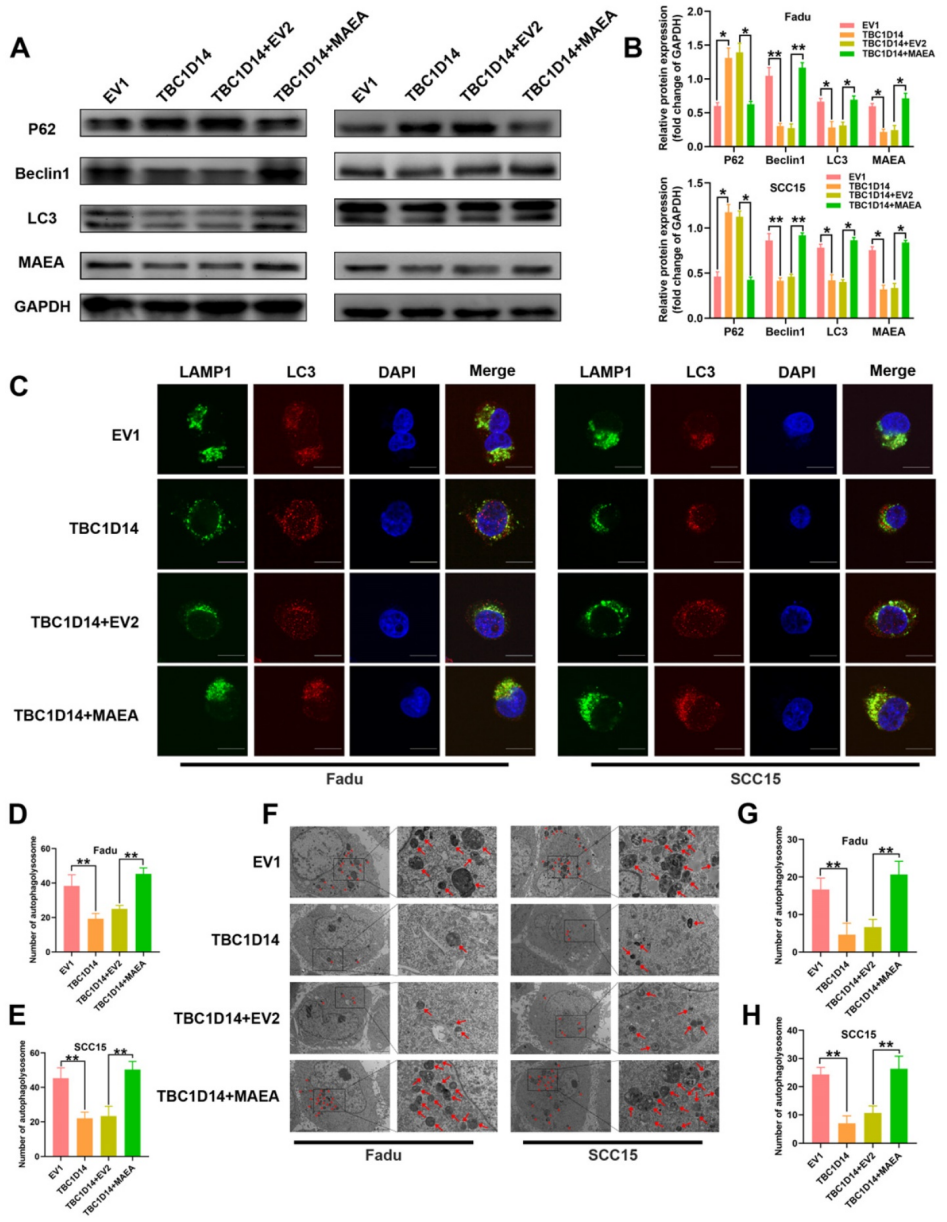
** TBC1D14 inhibited autophagy via downregulating MAEA.** (A-B) Protein level of autophagy markers was detected in TBC1D14-overexpressed cells transfected with empty or MAEA-coding plasmids by western blotting analysis (A). The relative protein expression was shown in the right-side histogram (B). (C-E) Immunofluorescence assay was performed to evaluated the co-localization of LC3 (green) and LAMP1 (red) in TBC1D14-overexpressed cells transfected with empty or MAEA-coding plasmid (C). Numbers of co-localized puncta (D-E) were counted with the help of ImageJ software. Scare bar: 10μm. (F-H) Autophagosomes were observed with the help of transmission electron microscope in TBC1D14-overexpressed cells transfected with empty or MAEA-coding plasmid (F). Red arrows indicated the representative autophagosomes. Counted numbers of autophagosomes were shown in right-side histograms (G-H). Scare bar: 5μm (x1500), 2μm (x5000). * *P*-value <0.05, **0.01. EV1: empty vector of TBC1D14. EV2: empty vector of MAEA.

As we have previously identified the negative correlation between TBC1D14 expression and MAEA both in vitro and in vivo, we next investigated whether TBC1D14 inhibited autophagy by suppressing MAEA. Protein levels of autophagy markers in TBC1D14-overexpressing HNSCC cells transfected with MAEA-coding plasmid or negative control plasmid were quantified. Western blotting analysis showed that TBC1D14 overexpression significantly downregulated Beclin1 protein level and LC3-II/LC3-I ratio but upregulated p62 protein level. However, these effects were reversed by MAEA overexpression (Fig. 7A-B). Results of the immunofluorescence assay to analyze changes in the colocalization of LC3 and LAMP1 showed that MAEA-overexpressing cells reversed the TBC1D14-dependent reduction of the merged yellow puncta (Fig. 7C-7E). TEM analysis also yielded the same conclusion (Fig. 7F-7G). Ultimately, these results further confirmed that TBC1D14 could downregulate the expression of MAEA to inhibit autophagy.

### TBC1D14 inhibited LNM in HNSCC in vivo

**Fig 8:**
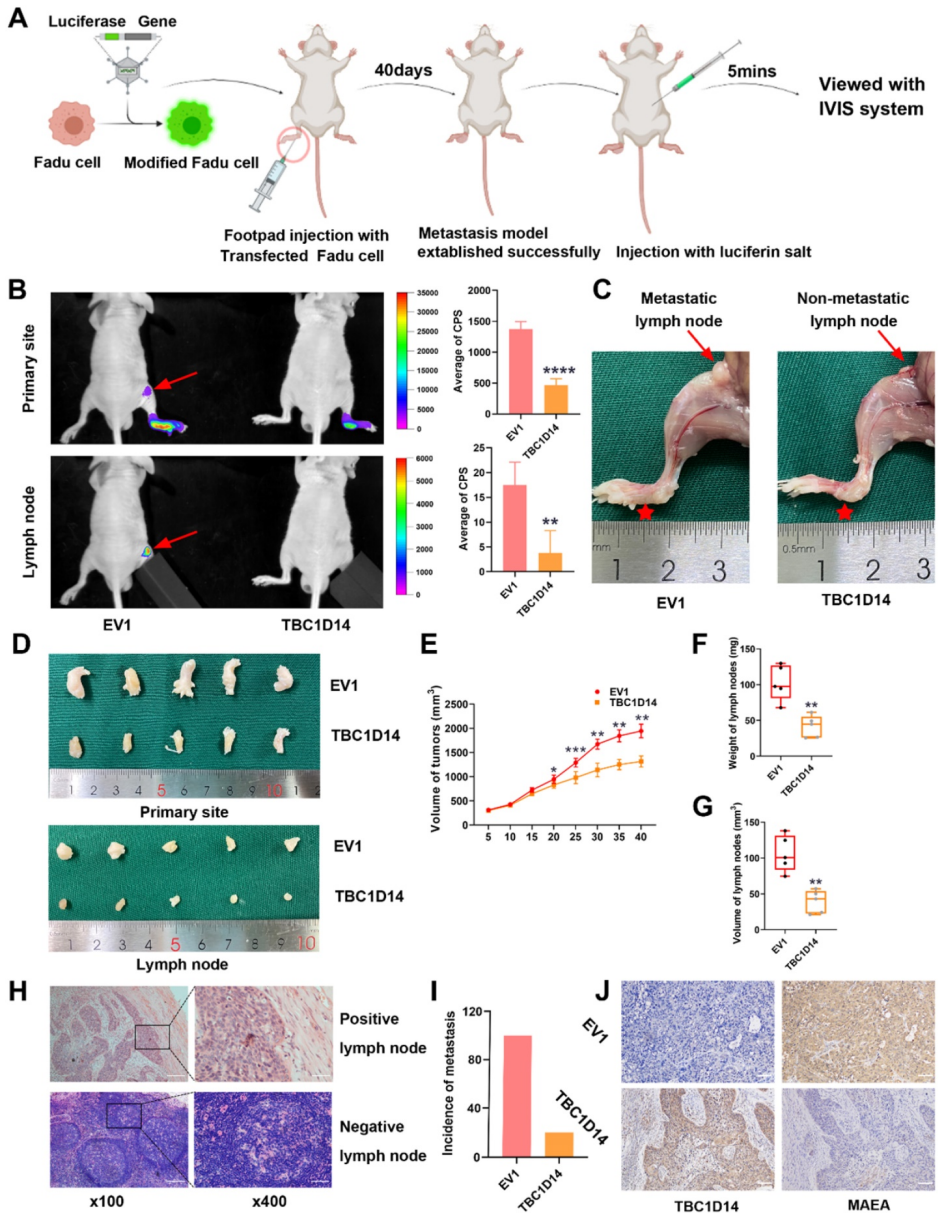
** TBC1D14 inhibited lymph node metastasis in vivo.** (A) The workflow of lymph node metastasis model. (B) Fadu cells with empty or TBC1D14 coding vector were injected into nude mice and visualized with IVIS system. Representative images of metastasis model were shown on the left side. Red arrows indicated the metastasis lymph node. Mean intensity of the fluorescent were shown on the right-side histography. (C) Representative anatomy maps of metastasis model. Red arrows indicated lymph node and red Pentagram indicated the primary-site tumors. (D-G) The primary-site tumors and lymph nodes from xenograft metastasis models were harvest at day 40 (D). Histograms represents the tumor volume (E) and lymph node volume (F) and weight (G). (H-I) Lymph nodes were examined by H&E staining to assess the status of metastasis. Representative metastatic lymph node was shown on the left side (H), and the incidence of metastasis was shown on the right-side graph (I). (J) Correlation between TBC1D14 and MAEA was assessed in the primary-site tumors by immunohistochemical staining. * *P*-value <0.05, **0.01; ***0.001; ****0.0001. IVIS: In Vivo Imaging System, EV1: empty vector of TBC1D14.

The role of TBC1D14 in LNM in vivo was studied by injecting 5×10^6^ Fadu cells stably overexpressing TBC1D14 or empty vector in nude mice to construct the LNM model as described in Fig. 8A. The IVIS visualization results showed that mice in the empty vector group had higher fluorescence intensity than those in the TBC1D14 overexpression group (Fig. 8B). The body weight of mice did not significantly change within 40 days (data not shown), but tumor volume, LN volume, and LN weight were lower in the TBC1D14 overexpression group than in the empty vector group (Fig. 8C-8G). These results were further validated by H&E staining of the LNs collected from mice in these two groups. Mice in the TBC1D14 overexpression group had a lower incidence of LNM than those in the empty vector group (Fig. 8H-8I; 5/5 vs. 1/5). The correlation between TBC1D14 expression and MAEA expression was studied in vivo by IHC staining, and the results were consistent with the in vitro results (Fig. 8J).

In summary, TBC1D14 suppresses LNM in HNSCC through inhibiting MAEA-mediated autophagy.

## Discussion

LNM is complex progress, which includes three main steps 45, 46. In the first step, cancer cells invade the extracellular matrix and migrate into the lymphatic vessels. Multiple factors, such as the migration and invasion ability, tumor-associated lymphangiogenesis, and escape from anoikis, determine the extent of invasion 29, 45, 47. After entering the lymphatic system, tumor cells further migrate into the lymphatic lumen and reach the draining LNs (also called Sentinel LNs) through the lymphatic stream. Intratumoral fluid pressure, immune escape, and chemokines are reported to mainly facilitate this progression 46, 48, 49. When malignant cells reach the draining site, they must survive against several stress conditions, including the lack of cellular connections, shortage of growth factors, and immune surveillance. The metastatic cells finally migrate into the LNs through the subcapsular sinus and form metastatic LNs 29, 45, 50.

Potential LM biomarkers were identified through whole-transcriptome sequencing of HNSCC primary-site tissues and then compared the results with data from TCGA and GEO datasets to eliminate bias caused by race and sample size. TBC1D14, a member of the TBC1 domain-containing family, was recognized as a co-DEG. TBC1Ds, characterized by the presence of the TBC (Tre2-Bub2-Cdc16) domain, regulate intracellular delivery to maintain homeostasis by inactivating Rab proteins 16, 51, 52. Recent studies in breast cancer revealed that TBC1D9 is a favorable prognostic biomarker, and TBC1D9 knockdown could facilitate tumor migration and growth both in vitro and in vivo 18, 20. Another study in epithelial ovarian cancer identified the tumor suppressor role of TBC1D16 21. In the present study, TBC1D14 exhibited an anti-metastatic effect in HNSCC. Patients without LNM had higher RNA (P = 0.0077) and protein (P = 0.0052) levels of TBC1D14. The five-year OS of HNSCC significantly benefited from the anti-metastatic role of TBC1D14 (P = 0.03). IHC staining also showed that patients without LNM had a lower percentage of strong staining (13/51 with vs. 17/23 without LNM; P < 0.001). These results suggested that TBC1D14 can potentially predict LNM and prognosis in HNSCC patients.

The invasion of the extracellular matrix by tumor cells is the initial and key step for LNM 45. Results from the in vitro assays confirmed that TBC1D14 could suppress the invasion and migration ability of HNSCC cells. The popliteal LNM model was applied to observe spontaneous LNM by injecting tumor cells into the footpad of nude mice 34, 53. As expected, the anti-metastatic role of TBC1D14 was further recognized in vivo.

Recently, autophagy was reported to facilitate tumor cell metastasis by activating various signaling pathways, such as TGFβ 54 and MAPK/ERK 55 pathways. The present analysis in HNSCC tissues with LNM also demonstrated the pro-metastatic role of autophagy in HNSCC. Moreover, several studies have revealed TBC1Ds could regulate autophagy in other tumors 56-58. Thus, it was hypothesized that TBC1D14 inhibits LNM by suppressing autophagy. As shown in the results, TBC1D14 overexpression suppressed autophagy by decreasing the protein levels of LC3-II and increasing the levels of p62. Immunofluorescence and TEM assays revealed that TBC1D14 overexpression could also inhibit autolysosome formation. Autophagy inhibitors reversed the enhanced migration of TBC1D14-KD cells. Taken together, these results suggested that TBC1D14 exhibits the anti-metastatic effect in HNSCC by inhibiting autophagy. Although the present findings are largely similar to previous findings in 293T cells 32, 33, the correlation between TBC1D14 and p62 was contrasting. In previous studies, TBC1D14 expression was only correlated with starvation-induced autophagy, and it did not change the protein levels of p62 in 293T cells. However, in the present study, TBC1D14 could inhibit autophagy without starvation stress, and its expression significantly positively correlated with p62 expression. The contrasting results could be because of the difference in cell lines. The huge demand for nutrients increases autophagy levels in tumor cells to fuel nearly all aspects of metabolism by degrading diverse substrates 59-61. Therefore, the correlation between TBC1D14 expression and autophagy in cancer cells without starvation induction seems plausible.

Label-free proteomics analysis recognized the correlation between MAEA, an autophagy promoter, and TBC1D14 expression. MAEA, also known as Erythroblast Macrophage Protein, is a novel type of E3 ubiquitin ligase. MAEA binds with RanBP9, Rmnd5, Armc8, and Twa1 to form a high-molecular-weight complex, called C-terminal to LisH (CTLH) complex, in both the nucleus and cytoplasm 62, 63. Previous studies have revealed MAEA could regulate AMPK and PI3K/ AKT pathways to promote autophagy 42-44, 64-67. While MAEA knock-out caused a maturation defect of autolysosomes 44. However, little is known about the correlation between TBC1D14 expression and MAEA. In vitro assays showed that TBC1D14 overexpression could decrease MAEA both at the RNA and protein levels, while TBC1D14 KD could reverse this inhibition. Subsequent IHC analysis further confirmed this negative correlation both in the nude mouse model and in HNSCC patients. Notably, MAEA could save TBC1D14-induced autophagy inhibition in vitro. Altogether, TBC1D14 inhibited autophagy by downregulating MAEA expression. Although the crucial role played by MAEA in TBC1D14-induced autophagy inhibition was recognized, the mechanism by which TBC1D14 downregulates MAEA expression in HNSCC is unclear, and subsequent experiments will be conducted.

This could be the first report of the pathogenic role of TBC1D14 expression. First, the anti-metastatic role of TBC1D14 in HNSCC was revealed, and the molecular mechanisms driving autophagy and LNM were uncovered. TBC1D14 functions as an LNM suppressor in HNSCC by downregulating MAEA expression to inhibit autophagy. Patients with a higher expression of TBC1D14 had a significantly better five-year OS. These findings provide new insights into the study of novel LNM biomarkers and underlying molecular mechanisms in HNSCC.

## Supplementary Material

Supplementary figure and tables.

## Figures and Tables

**Table 1:** The correlation between TBC1D14 protein levels and clinical characteristics of HNSCC patients (n=74)

	TBC1D14 expression		χ2 value	P value
	**Positive**	**Negative**		
**Gender**			1.487	0.223
**Male**	29	44		
**Female**	1	0		
**Age (y)**			0.214	0.624
**>60**	16	26		
**≤60**	14	18		
**Alcohol**			1.162	0.281
**Yes**	19	33		
**No**	11	11		
**Smoking**			0.008	0.931
**Yes**	25	37		
**No**	5	7		
**Pathological T stage**			0.293	0.588
**T1+T2**	7	8		
**T3+T4**	23	36		
**Lymph node metastasis**			15.419	<0.001^***^
**Positive**	13	38		
**Negative**	17	6		
**Pathological N stage**			15.764	0.001^***^
**N0**	17	6		
**N1**	2	9		
**N2**	8	22		
**N3**	3	7		
**Extranodal extension**			4.121	0.042^*^
**Positive**	5	17		
**Negative**	25	27		
**Differentiation**			0.370	0.831
**Poorly**	7	13		
**Moderate**	16	21		
**Well**	7	10		

* *P*-value <0.05, *** 0.001.

## Abbreviations

HNSCC: head and neck squamous cell carcinoma
OS: overall survival
LNM: lymph node metastasis
LN: lymph node
TCGA: The Cancer Genome Atlas
GEO: Gene Expression Omnibus
TBC1D14: TBC1 domain family member 14
DEG: differentially expressed gene
MAEA: macrophage erythroblast attacher
LC3: light chain 3
ATG5: autophagy-related 5
IF: Immunofluorescence
TME: Transmission electron microscopy
IHC: Immunohistochemistry
ENE: extranodal extension
CQ: chloroquine
BA: bafilomycin A1
