# Supplementary material for: TBC1D14 inhibits autophagy to suppress lymph node metastasis in head and neck squamous cell carcinoma by downregulating macrophage erythroblast attacher
Source: Int J Biol Sci. 2022 Feb 7;18(5):1795–812. doi: 10.7150/ijbs.68992 (PMC8935227; doi:10.7150/ijbs.68992)
Supplement: Supplementary file 1 — Supplementary figure and tables. [file ijbsv18p1795s1.pdf]

Supplementary Table. S1 Sequences of primers used for qRT-PCR analysis and shRNA constructs.

	**Name**	**Sequence**
**Primers**	TBC1D14 Forward (5'-3')	TGGTGGTTCAGGCCAAAAAG
	TBC1D14 Reverse (3'-5')	GAGCACAGCGTTTCCAATGC
	GAPDH Forward (5'-3')	CAGCGACACCCACTCCTC
	GAPDH Reverse (3'-5')	TGAGGTCCACCACCCTGT
	MAEA Forward (5'-3')	AACGAGAACAATCCGCCCAT
	MAEA Reverse (3'-5')	TTCTTTGGTTCTCGGGCACA
**shRNA**	ShTBC1D14	CCGAAATTATTGCGCATT

Supplementary Table. S2 Details of the antibodies and agents.

	Name	Dilution	Companay	Catalog Number
Primary antibody	TBC1D14	1:2000 (WB)<br>1:100 (IHC)	Abcam (Cambridge, USA)	ab235544
	GAPDH	1:2000 (WB)	Abcam (Cambridge, USA)	ab8245
	LC3	1:2000 (WB)<br>1:500 (IF)	Abcam (Cambridge, USA)	ab192890
	SQSTM1/p62	1:2000 (WB)	Abcam (Cambridge, USA)	ab207305
	Beclin1	1:2000 (WB)	Abcam (Cambridge, USA)	ab207612
	ATG5	1:1000 (WB)	Cell Signaling Technology (Danvers, USA)	#9980
	MAEA	1:1000 (WB)	Proteintech (Chicago, USA)	28363-1-AP
	LAMP1	1:500 (IF)	Cell Signaling Technology (Danvers, USA)	#15665
Secondary antibody	Goat anti-rabbit IgG (HRP)	1:5000 (WB)	Abcam (Cambridge,	ab6721

			USA)	
	Goat anti-rabbit IgG (Alexa Fluor® 555 Conjugate)	1:250 (IF)	Abcam (Cambridge, USA)	Ab150078
	Goat anti-mouse IgG (Alexa Fluor® 647 Conjugate)	1:250 (IF)	Cell Signaling Technology (Danvers, USA)	#4410
Agents	Chloroquine	10 μm/ml	MedChemExpress (Monmouth Junction, USA)	HY-17589A
	Bafilomycin A1	100nm/ml	MedChemExpress (Monmouth Junction, USA)	HY-100558

WB: western blotting analysis; IHC: Immunohistochemistry staining; IF: immunofluorescence assay.

Supplementary Table. S3 Details of co-differentially expressed genes.

Name	RNA-SEQ	TCGA	GEO
ABCA3*	up	up	up
ALKBH8	up	down	down
ANXA6*	up	up	up
AP4E1*	down	down	down
ARHGAP4*	up	up	up
AURKB*	up	up	up
BCAT2*	up	up	up
CKLF	down	up	up
CLCA2*	down	down	down
DSC1*	down	down	down

DSC3*	down	down	down
DSG1*	down	down	down
EI24*	down	down	down
EVPL*	down	down	down
GABRP*	up	up	up
GALNT1*	down	down	down
GPR1*	down	down	down
HOXB13*	up	up	up
HVCN1*	up	up	up
IGFL1*	down	down	down
JMJD6*	up	up	up
KRT6C*	down	down	down
KRT75*	down	down	down
LGI2*	up	up	up
MAD2L2*	up	up	up
MCM5	down	up	up
MLF1*	up	up	up
NECAP2	down	up	up
PFDN5	down	up	up
PLA2G4C*	up	up	up
RELB*	up	up	up
SALL1*	down	down	down

SERPINB3*	down	down	down
SIPA1L2	up	down	down
SRL	up	down	up
TACC2*	down	down	down
TBC1D14*	down	down	down
TESC*	up	up	up
TMEM154*	down	down	down
TMEM45B*	down	down	down
TRIM29*	down	down	down
UBD*	up	up	up
VCAM1*	up	up	up
ZMYND11	up	down	down
ZWILCH	down	up	down

* co-differentially expressed genes with same tendency in all 3 cohorts.

Supplementary Figure. S1 TBC1D14 expression was higher in normal tissues than in tumors.

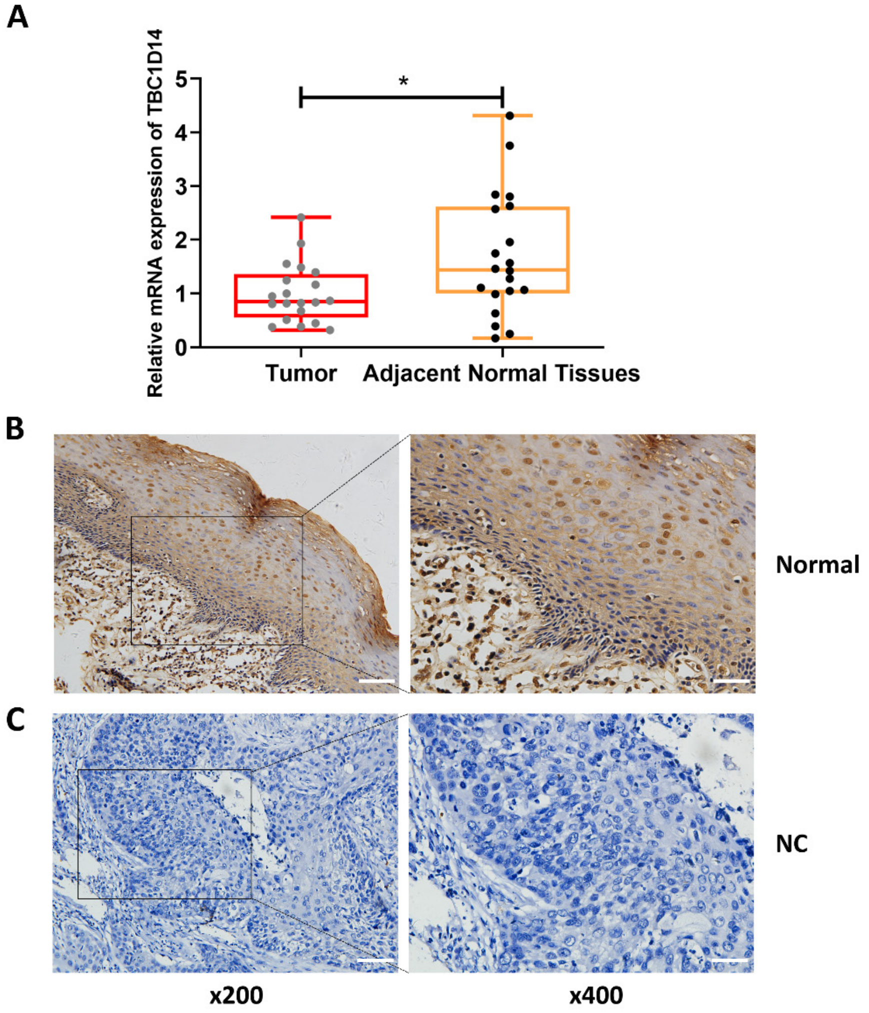

A. qRT-PCR analysis was conducted in HNSCC tumor tissues and compared adjacent normal tissues; B-C. Immunohistochemical staining was performed in adjacent normal tissues of HNSCC (C), tissues in control group was staining with PBS instead of primary antibody (D).

* *P*-value <0.05.
